# Rare clinical presentations of hyper‐IgE syndrome in a patient with dental abnormalities: A case report

**DOI:** 10.1002/ccr3.4692

**Published:** 2021-08-23

**Authors:** Marzieh Heidarzadeh Arani, Atena Ramezanali Yakhchali, Mohammad Gharagozlou, Sepideh Darougar, Zahra Chavoshzadeh, Mahnaz Jamee, Hossein Motedayyen

**Affiliations:** ^1^ Department of Pediatrics Kashan University of Medical Sciences Kashan Iran; ^2^ Shahid Beheshti University of Medical Sciences and Health Services Tehran Iran; ^3^ Department of Allergy and Clinical Immunology Children's Medical Center Tehran University of Medical Sciences Tehran Iran; ^4^ Department of Pediatrics Tehran Medical Sciences Branch Islamic Azad University Tehran Iran; ^5^ Immunology and Allergy Department Mofid Children's Hospital Shahid Beheshti University of Medical Sciences Tehran Iran; ^6^ Pediatric Nephrology Research Center Research Institute for Children's Health Shahid Beheshti University of Medical Sciences Tehran Iran; ^7^ Autoimmune Diseases Research Center Kashan University of Medical Sciences Kashan Iran

**Keywords:** allergic reactions, dental abnormality, Hyper‐IgE syndrome, recurrent infections, signal transducer and activator of transcription 3

## Abstract

Asthma and anaphylaxis are two atypical presentations of hyper‐IgE syndrome (HIES). Early diagnosis and management of HIES can improve quality of life of patients through minimizing orthodontic problems and other complications related to this disorder.

## Graphical abstract

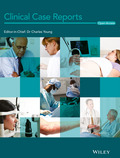

## 1 INTRODUCTION

Autosomal dominant hyper‐IgE syndrome (AD‐HIES), also known as Buckley or Job's syndrome, is a rare multisystem disorder with immunologic and nonimmunologic characteristics.^1^ Annual incidence of this disorder is estimated at one case per 1,000,000 individuals.^2^ It is largely associated with heterozygous dominant‐negative mutations in the *signal transducer and activator of transcription 3* (*STAT3*) gene. The most frequent clinical manifestations among AD‐HIES patients are eczematoid rashes, staphylococcal skin abscesses, pruritic dermatitis, dental abnormalities, oral cavity defects, mucocutaneous candidiasis, connective tissue defects, and high serum IgE level.^3^, ^4^, ^5^, ^6^ Some clinical problems are rarely observed in patients with AD‐HIES, including allergic rhinitis, asthma, urticaria, and anaphylaxis.^7^, ^8^

In this case report, we reported asthma and anaphylaxis as two atypical presentations in an AD‐HIES patient suffering from dental abnormalities, eczema, and recurrent sinusitis.

## 2 CASE HISTORY

**FIGURE 1:**
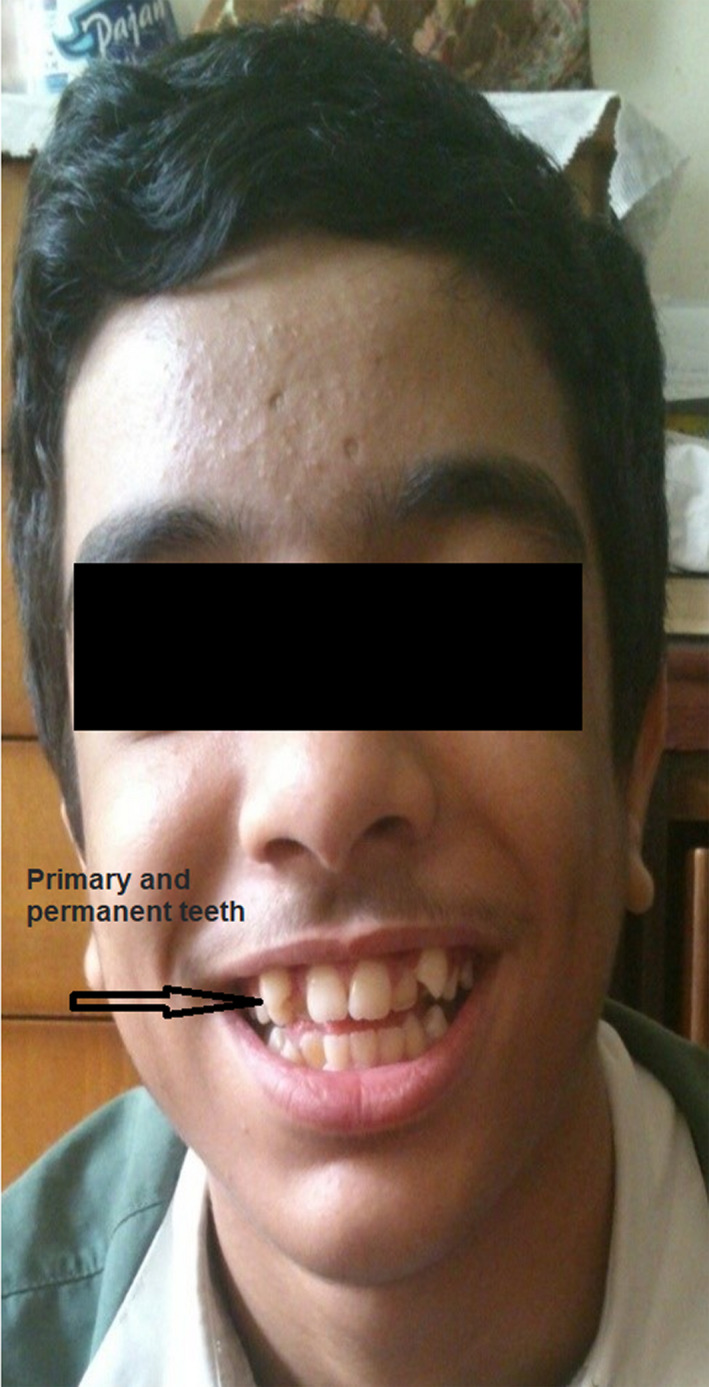
Dental complications in a patient with AD‐HIES. Primary teeth were retained accompanied by eruption of permanent teeth (double‐rowed teeth)

The case was a 14‐year‐old boy. He was the third child of unrelated parents. Erythematous papules and vesicles were observed on the glans penis after circumcision procedure. At 6 months of age, he was referred to the allergy and immunodeficiency clinic of Kashan Shahid Beheshti hospital, Iran, due to facial dermatitis (eczema). The result of skin prick test indicated hypersensitivity reactions to milk, egg, and wheat. Atopic dermatitis was diagnosed. He was treated according to guidelines for the treatment of atopic eczema.^9^ Foods containing eggs, milk, and wheat were also eliminated from daily diets. A replacement diet was designed to provide substitutes for the eliminated foods for maintenance of balanced nutrition.^10^ At 18 months of age, our case suffered from cough, dyspnea, and wheezing, which their severity increased with age. Clinical evaluations revealed that our patient suffered from asthma. He was treated based on the 2019 Global Initiative for Asthma (GINA) guidelines.^11^ At 10 years of age, acute asthma attacks and eczema were accompanied by recurrent sinusitis. These complications were, respectively, treated with inhaled corticosteroids and antibiotic therapy (amoxicillin/clavulanic acid 625 mg BD). At 12 years of age, our patient showed some clinical symptoms related to anaphylaxis, which were managed according to guidelines for anaphylaxis treatment.^12^ His growth and weight were normal. However, retention of primary teeth was accompanied by ectopic eruption of permanent teeth (Figure 1). Based on these findings, an immunodeficiency disorder was suspected. Some laboratory tests were used to assess his immunological situation (Table 1). Laboratory blood tests revealed a significant increase in serum IgE level (1320 IU/ml). This finding along with our previous observations suggested that our patient might suffer from hyper‐IgE syndrome (HIES). The patient was genetically assessed to determine a possible defect in the *STAT3* gene. The genetic analysis indicated that he had a heterozygous mutation in *STAT3* leading to an amino acid change (V637M) in the SH2 domain through a nucleotide exchange of 1909 G to A in the exon 21. This genetic change is considered as a major cause of AD‐HIES.

**TABLE 1:** Laboratory characteristics of a patient with AD‐HIES

	Cell numbers or values	Total counted cells or normal ranges
White blood cell: 7100	7.1 × 10^9^/L (polymorphonuclear cells: 75%, lymphocytes:24%)	3.5–12 × 10^9^/L
Hemoglobin	150 g/L	130–170 g/L
Platelet	280 × 10^9^/L	150–450 × 10^9^/L
IgA	57 mg/dl	25–154 mg/dl
IgM	48 mg/dl	37–224 mg/dl
IgG	889 mg/dl	386–1470 mg/dl
IgE	1320 IU/ml	Children (6–15 years): <150 IU/ml

Regarding that our patient suffered from asthma and was dissatisfied with further evaluations, he was treated with budesonide/formoterol (Symbicort Turbuhaler) 160/4.5 mg/inhalation one puff twice daily. Our patient is currently alive and in acceptable health condition.

## 3 DISCUSSION

Signal transducer and activator of transcription 3 is a critical regulator of multiple processes, including cellular proliferation, survival, differentiation, fetal development, cancer, wound healing, angiogenesis, autoimmunity, and inflammation.^6^ Impaired function of this regulator is related to different complications such as dental abnormalities and connective tissue defects.^6^ In this case report, we investigated a boy with acute asthma attacks, eczema, anaphylaxis, recurrent sinusitis, and dental abnormalities.

There are some reports pointing to dental complications in AD‐HIES.^13^ It is revealed that approximately 72% of patients with HIES have retention in primary teeth.^14^ O'Connell et al. conducted a study on 34 patients with HIES and indicated that 75% of subjects older than 7 years had eruption problems in the permanent dentition, in the form of prolonged retention of primary teeth or the need for extraction of primary teeth.^15^ In agreement with our findings, there are some reports indicating patients with HIES have prolonged retention of primary dentition accompanied by delayed eruption of permanent teeth.^16^

Although abnormal dentition is a common clinical feature of AD‐HIES, mechanism(s) involved in this complication is not well identified yet. O'Connell et al. suggested that the unusual persistence of Hertwig's epithelial root sheath on the root of a primary tooth may be considered as a mechanism related to delayed resorption in patient with HIES.^16^ Another possible mechanism may correlate to STAT3 functions, which as a transcription factor play indispensable roles in signaling pathways of various cytokines such as IL‐2, IL‐6, IL‐10, IL‐12, IL‐15, IL‐21, IL‐23, and IL‐27.^17^, ^18^, ^19^ Among these cytokines, IL‐6 has a positive role in bone resorption through inducing osteoclastogenesis.^20^, ^21^ Thus, impaired STAT3 signaling may disrupt the role of IL‐6 in osteoporosis and thereby contribute to retention of primary teeth in AD‐HIES. In line with this notion, Grimbacher et al. proposed that delayed resorption of primary teeth in HIES may result in ineffective inflammatory responses and the formation of pneumatoceles. Furthermore, it is suggested that dental root resorption is associated with the activation of osteoclasts and/or macrophages by cytokines.^16^ Nevertheless, it is worthy that future studies will be designed to clarify whether dental abnormalities in patients with AD‐HIES are related to defect in cytokine functions or other immunologic and nonimmunologic mechanisms.

Asthma and anaphylaxis are two atypical presentations of AD‐HIES. According to the literature, some allergic manifestations such as allergic rhinitis, asthma, urticaria, and anaphylaxis are rarely observed in patients with AD‐HIES, despite their high serum IgE concentration. In a study regarding effect of STAT3 mutation on serum IgE level in asthmatic children, it is revealed that this genetic variation had no impact on the elevated IgE level.^7^ Moreover, there are some reports showing no correlation of STAT3 expression with mild and severe refractory asthma.^8^ However, we observed some clinical manifestations pointing to an allergic background in our case, including neonatal dermatitis with positive skin prick test to milk, egg, and wheat, a favorable response to an elimination diet, a history of anaphylaxis, and recurrent asthma exacerbations.

Taken together, our findings indicate that clinical evaluations of some atypical presentations, such as asthma and anaphylaxis, along with immunological and genetic analyses can be helpful to early diagnosis and management of AD‐HIES, which can minimize later orthodontic problems and other potential complications associated with this syndrome. Therefore, patients suffering from allergic and infectious diseases, autoimmune disorders, and dental abnormalities should be investigated by clinical, immunological, and genetic tests to determine possible mutations in *STAT3* gene.

## CONFLICT OF INTEREST

None declared.

## AUTHOR CONTRIBUTIONS

MHA and ARY contributed to the conceptualization, data curation, supervision, and writing the original draft. MG, SD, ZC, and MJ and HM reviewed and edited the final manuscript. All authors read and approved the final manuscript.

## ETHICAL APPROVAL

Written informed consent was obtained from parents for the clinical data and photographs of their child to be published. This study is approved by the Ethics Committee of Kashan University of Medical Sciences (IR.KAUMS.MEDNT.REC.1400.028).

## Data Availability

All data generated or analyzed during the study are included in this published case report.
